# Prediction of recovery after revascularization in chronic Coronary Total Occlusion (CTO) patients. Adenosine or low-dose dobutamine stress with LGE CMR: which is the best combination?

**DOI:** 10.1186/1532-429X-17-S1-P114

**Published:** 2015-02-03

**Authors:** Antoine Gerbay, Emile Youssof, Marco Vola, Charles de Bourguignon, Alexis Cerisier, Magalie Viallon, Karl Isaaz, Pierre Croisille

**Affiliations:** Radiology, CHU Saint-Etienne, Université J.Monnet, Université Lyon, CREATIS UMR CNRS 5220 INSERM U1044, Saint-Etienne, France; Cardiology, CHU Saint-Etienne, Université J.Monnet, Université Lyon, Saint-Etienne, France; Surgery, CHU Saint-Etienne, Université J.Monnet, Université de Lyon, Saint-Etienne, France

## Background

Recanalisation of chronic total occlusion (CTO = coronary occlusion > 3 months with a TIMI flow grade 0), is one of the most challenging PCI procedure with specific complications. Eventhough LGE-CMR is routinely used to assess viability, it does not assess ischemic status or inotropic reserve of regions surrounding scar lesions that are particularly at risk of jeopardy in patients with collateralized myocardium. If adenosine stress perfusion (Adeno) has an established high diagnostic accuracy for the detection of CAD, its specific value in the scope of chronic ischemia compared to LGE CMR imaging only or combined to low-dose dobutamine (LDD) is uncertain. The aim of this study was therefore to evaluate which best combination could improve prediction of functional recovery after revascularization of chronic total occlusion (CTO).

## Methods

58 patients were prospectively enrolled with CMR at baseline. 37 patients were revascularized and all underwent CMR at 6 months follow up with a follow-up coronary angiography within 5±3 days. All CMR (1.5T) examination included: rest cine study, LDD stress with cine imaging at 5,10 ±15γ (criteria=10% DP increase), Adeno stress perfusion and LGE acquisitions. Coronary CTO were performed using antegrade or retrograde techniques with drug eluting stent in all procedures. LV function et volumes were quantified, contractile reserve under LDD was assessed by visual analysis on a semi-quantitative scale, as well as stress perfusion that determined the extent of of perfusion defects without scar lesion. A logistic regression model was used to compare the imaging strategies (LGE alone, LGE+Adeno, LGE+LDD) and to evaluate incremented prediction of contractile function recovery in CTO-dependent segments.To compare the adequacy of various fitted logistic models, the areas of the corresponding receiver operating characteristic (ROC) curves where also obtained.

## Results

After revascularisation mean ESV decreased from 40,5 ml/m^2^ (+/-16,7) to 36,2 ml/m^2^ (+/-13,8) (p=0,06). EDV remained unchanged from 86,3 ml/m^2^ (+/-17,8) to 84,0 ml/m^2^ (+/-16,2) (p=0,39). Revascularized patients had a significant LVEF improvement (p=0,04).

592 segments were studied among which 214 in CTO dependent territories, with 109 dysfunctional segments at rest. In the multivariate model, transmurality of necrosis was the strongest predictor for recovery with a chance of recovery reaching an OR=3.29 that was superior to to Adenosine (OR=1.69) and dobutamine (OR=1.38). Compared to LGE only (Az:0.79), the addition of functional stress tests in the prediction model lead to an improvement of performance for both, but that was superior for Adeno (Az:0.85) compared to dobutamine (Az:0.82), with a near significant difference for Adeno only (p=0.06).

## Conclusions

In a CTO population, the combination of LGE + Adenosine stress perfusion improves prediction of recovery in CTO-dependent segments as determined 6 months after revascularization.

## Funding

N/A.

